# Self‐Assembled Surfactant‐Polyoxovanadate Soft Materials as Tuneable Vanadium Oxide Cathode Precursors for Lithium‐Ion Batteries

**DOI:** 10.1002/anie.202216066

**Published:** 2023-02-09

**Authors:** Rory C. McNulty, Keir Penston, Sharad S. Amin, Sandro Stal, Jie Yie Lee, Mario Samperi, Lluïsa Pérez‐García, Jamie M. Cameron, Lee R. Johnson, David B. Amabilino, Graham N. Newton

**Affiliations:** ^1^ Nottingham Applied Materials and Interfaces (NAMI) Group School of Chemistry University of Nottingham Nottingham NG7 2TU UK; ^2^ The Faraday Institution, Quad One Harwell Science and Innovation Campus Didcot OX11 0RA UK; ^3^ GSK Carbon Neutral Laboratories for Sustainable Chemistry School of Chemistry University of Nottingham Nottingham NG7 2TU UK; ^4^ CNR-ITAE Via Salita Santa Lucia Sopra Contesse 5 98126 Messina Italy; ^5^ Departament de Farmacologia i Química Terapèutica Universitat de Barcelona Av. Joan XXIII, 27–31 08028 Barcelona Spain; ^6^ Institut de Ciència de Materials de Barcelona (ICMAB) Consejo Superior de Investigaciones Científicas Campus Universitari de Bellaterra 8193 Cerdanyola del Vallès Spain

**Keywords:** Electrochemistry, Energy Storage, Metal Oxide, Polyoxometalate, Self-Assembly

## Abstract

The mixing of [V_10_O_28_]^6−^ decavanadate anions with a dicationic gemini surfactant (***gem***) leads to the spontaneous self‐assembly of surfactant‐templated nanostructured arrays of decavanadate clusters. Calcination of the material under air yields highly crystalline, sponge‐like V_2_O_5_ (***gem*** **‐V_2_O_5_**). In contrast, calcination of the amorphous tetrabutylammonium decavanadate allows isolation of a more agglomerated V_2_O_5_ consisting of very small crystallites (***TBA*** **‐V_2_O_5_**). Electrochemical analysis of the materials’ performance as lithium‐ion intercalation electrodes highlights the role of morphology in cathode performance. The large crystallites and long‐range microstructure of the ***gem*** **‐V_2_O_5_** cathode deliver higher initial capacity and superior capacity retention than ***TBA*** **‐V_2_O_5_**. The smaller crystallite size and higher surface area of ***TBA*** **‐V_2_O_5_** allow faster lithium insertion and superior rate performance to ***gem*** **‐V_2_O_5_**.

## Graphical abstract

Decavanadate anions self‐assemble with a dicationic surfactant to form ordered arrays which, when calcined, yield highly crystalline V_2_O_5_. In contrast, calcination of alkylammonium decavanadate leads to smaller V_2_O_5_ crystallites. The systems show contrasting, topology‐dependent performance as cathode materials in Li‐ion batteries, offering a route to the controlled preparation of V_2_O_5_ electrode materials that balance energy and rate performance.

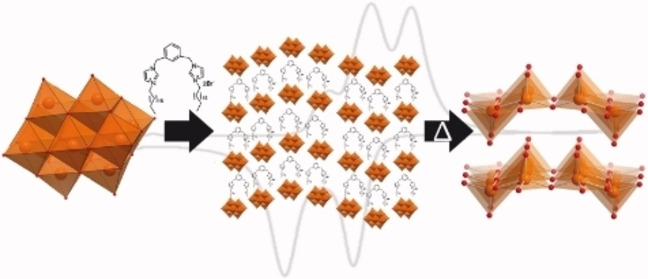

Polyoxometalates (POMs; anionic oxo‐clusters of the group V and VI metals), are a class of soluble and (relatively) stable molecular fragments of bulk metal oxides that can act as precursors for the atomistically controlled fabrication of nanostructured metal oxides.^[1]^ Their physical properties (solubility, stability, redox activity etc.) can be effectively tuned by the nature of their charge balancing counter cations.[^2^, ^3^, ^4^] For example, tetrabutylammonium (TBA) salts of POMs are typically soluble in polar organic solvents and have been widely applied as photoredox catalysts for organic oxidation reactions.^[5]^ Similarly, use of long chain alkylammonium or phosphonium cations allows preparation of POM‐based ionic liquids,^[6]^ and use of rigid aromatic or amphiphilic cations can serve as structure‐directing agents, leading to the formation of highly ordered soft organic–inorganic “hybrid” materials.[^7^, ^8^, ^9^, ^10^, ^11^] Thermal treatment of such species can lead to the condensation of neighbouring POM molecules to yield extended bulk metal oxides, where nanostructuring and the resulting topography is impacted by the nature of the precursor.[^1^, ^12^]

Bulk V_2_O_5_ has been widely studied as an ion intercalation material, thanks to its layered graphite‐like structure which was originally theorised to facilitate fast, reversible ion transfer.^[13]^ Several factors complicate this theoretical performance, most related to diffusion limitations and low electron mobilities,[^14^, ^15^] however nanostructuring is seen as a key mitigation strategy to control diffusion pathlengths and modify the electronic structure of the cathode material.^[16]^ The ease of fabrication, abundance of raw materials, low cost, and moderate lithium intercalation capacity of nanostructured V_2_O_5_ materials has generated great interest for their development as a lithium‐ion battery intercalation material.[^15^, ^16^, ^17^] The promising electrochemical performance provides motivation for materials development, with a theoretical single lithium insertion capacity (147 mAh g^−1^) comparable to that of the ubiquitous LiFePO_4_ (LFP, 170 mAh g^−1^).[^18^, ^19^] Previous reports have demonstrated the relationship between nanostructure and electrochemical performance, with synthesis of porous and hierarchical structures delivering considerable performance enhancement.[^14^, ^16^, ^17^, ^18^, ^20^]

Most current approaches to the synthesis of V_2_O_5_ rely on the treatment of simple molecular vanadate precursors under a range of conditions (hydro/solvothermal, pyrolysis, electrospinning, exfoliation, etc.).[^21^, ^22^, ^23^, ^24^, ^25^, ^26^] Though an effective means to generate V_2_O_5_, these fabrication methods often lack precise control over the structure and morphology of the product and tend to rely on a “trial and error” approach rather than targeted synthetic design. In light of this situation, recent examples have explored the use of structure‐directing agents to generate hierarchical V_2_O_5_ nanostructures that exhibit enhanced stability and performance.[^27^, ^28^, ^29^, ^30^] Continued development of new methods for the controlled synthesis of nanostructured V_2_O_5_ materials has the potential to unlock advances in both the reliable production and performance of these materials.

Here, we show that combination of decavanadate ([V_10_O_28_]^6−^) clusters with structure‐directing cationic gemini surfactants leads to the formation of nanostructured soft‐materials. In contrast, a tetraalkylammonium salt of the clusters exhibited no significant long‐range ordering. Pyrolysis of the ordered material yields V_2_O_5_ nanorods assembled into “sponge‐like”, microporous aggregates with the surfactant guiding the formation of well‐ordered and defined crystalline nanorods. Pyrolysis of the tetraalkylammonium salt leads to isolation of a disordered bulk material consisting of smaller V_2_O_5_ crystallites with a higher degree of sintering than the gemini surfactant‐derived sample. Electrodes prepared from the ordered V_2_O_5_ are shown to exhibit greater lithium intercalation capacities than those based on the amorphous analogue. We demonstrate the role that V_2_O_5_ nanostructure plays on the performance of lithium‐ion cells.

The dicationic gemini surfactant 1,3‐bis[(3‐octadecyl‐1)imidazolio)methyl]benzene (***gem***),^[31]^ was selected as a structure‐directing agent due to its known propensity to strongly bind a range of anions[^32^, ^33^] and its ability to form a range of self‐assembled supramolecular aggregates (e.g. hydrogels, nanofibers or stabilised metal nanoparticles) thanks to its amphiphilic character.[^33^, ^34^, ^35^] The decavanadate anion [V_10_O_28_]^6−^ (**V_10_**) is among the most commonly studied polyoxovanadate species owing to its relatively simple synthesis and useful photo‐ and redox‐properties.[^36^, ^37^] Furthermore, its limited thermal stability (in comparison to W‐based clusters, for example) provides a straightforward route to the relatively low temperature thermal preparation of phase‐pure vanadium oxides.^[38]^ (Further details of synthesis and characterisation of the precursors can be found in the Supporting Information).

Simple combination of the structure‐directing gemini surfactant (***gem***) and K_6_[V_10_O_28_] (**K‐V_10_**) via ion metathesis was used to prepare the organic–inorganic hybrid precursor. This was accomplished mixing an ethanolic solution of the dibromide salt of ***gem*** with an aqueous solution of **K‐V_10_** (Scheme 1). In a typical synthesis, 1 molar equivalent of POM was combined with 1.5 equivalents of ***gem*** in a mixed water/ethanol solution (H_2_O : EtOH; 9 : 1 *v/v*). The resulting suspension was vacuum filtered and then washed with EtOH, followed by H_2_O, to yield an orange powder corresponding to *(gem*)_3_[V_10_O_28_] (***gem*** **‐V_10_**), as characterized by FT‐IR, TGA and elemental analysis (Figure S1). Transmission electron microscopy (TEM) revealed the ***gem*** **‐V_10_** powder to consist of aggregated particles (ca. 50–100 nm in diameter) (Scheme 1). Note that a similar morphology was reported recently for an analogous composite material,^[39]^ which the authors attribute to an ionic self‐assembly process, whereby the surfactant‐associated POM clusters condense through non‐covalent interactions to form larger aggregated nanostructures. Here, high magnification images captured using high‐resolution TEM (HR‐TEM) imaging of the ***gem*** **‐V_10_** particles shows that each of these nodular particles contain a distinct lamellar structure with an approximate interlayer spacing of 2–3 nm (Scheme 1). This distance corresponds to the separation of anionic POM clusters by cationic ***gem*** molecules into ordered layers and suggests that, as hoped, the combination of ***gem*** and **V_10_** leads to spontaneous formation of a supramolecular nanostructure. The tetrabutylammonium salt of the [V_10_O_28_] anion, (TBA)_3_[H_3_V_10_O_28_] (**TBA‐V_10_**) was synthesised as a “non‐specific” control material and characterised in a similar manner (Further details can be found in the Supporting Information). TEM analysis (Scheme 1) confirms that the material obtained by precipitation from an ethanolic solution consists of microparticles with no apparent periodic nanostructuring.

**Scheme 1:**
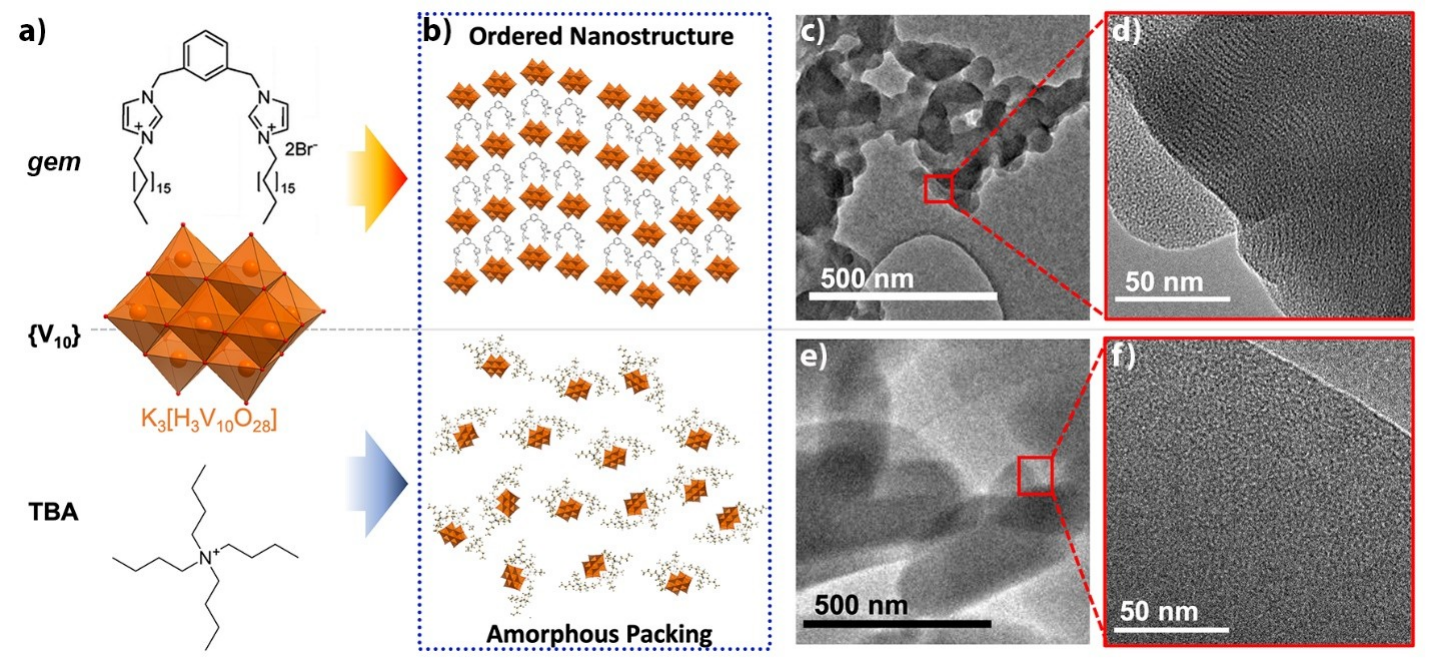
a) Simplified synthetic scheme of ***gem*** **‐V_10_** and ***TBA*** **‐V_10_**, and b) an impression of the potential ordering in the solid state of ***gem*** **‐V_10_** (top) and ***TBA*** **‐V_10_** (bottom). c)–f) HR‐TEM images of the two materials illustrate the ordering of the decavanadate clusters in ***gem*** **‐V_10_** (note the dark lines of clusters in (d)) and the apparently amorphous nature of ***TBA*** **‐V_10_** in (f).

The use of ***gem*** **‐V_10_** as a supramolecular precursor for the formation of bulk vanadium oxides was then explored via calcination of the precipitate in an open tube‐furnace (i.e. under air). A range of calcination temperatures and times were explored, with optimal results (in terms of product consistency and phase purity) identified when ***gem*** **‐V_10_** was calcined at 550 °C for 2 hours. These conditions resulted in the formation of an orange‐brown powder (***gem*** **‐V_2_O_5_**) which was unambiguously identified by powder X‐ray diffraction (PXRD) as phase‐pure, orthorhombic V_2_O_5_ and supported by energy dispersive X‐ray spectroscopy (EDX) analysis (Figures S2, S3&S7). The detailed morphology of ***gem*** **‐V_2_O_5_** was subsequently analysed using scanning electron microscopy (SEM). The micrographs showed that the nodular, semi‐amorphous precursor ***gem*** **‐V_10_** is converted into a highly crystalline “sponge‐like” material comprising rod‐like V_2_O_5_ crystallites typically 1–5 μm in length and between 0.5–2 μm in diameter (Figure 1a–c). The formation of these crystallites is also found to be controllable, where their size strongly depends on the heating temperature (Figure S4). Increasing calcination temperature from 550 °C to 600 °C to leads to the formation of large rod‐like crystals (approaching 20 μm in length and 1–2 μm wide) (see Supporting Information for further detail).

**Figure 1:**
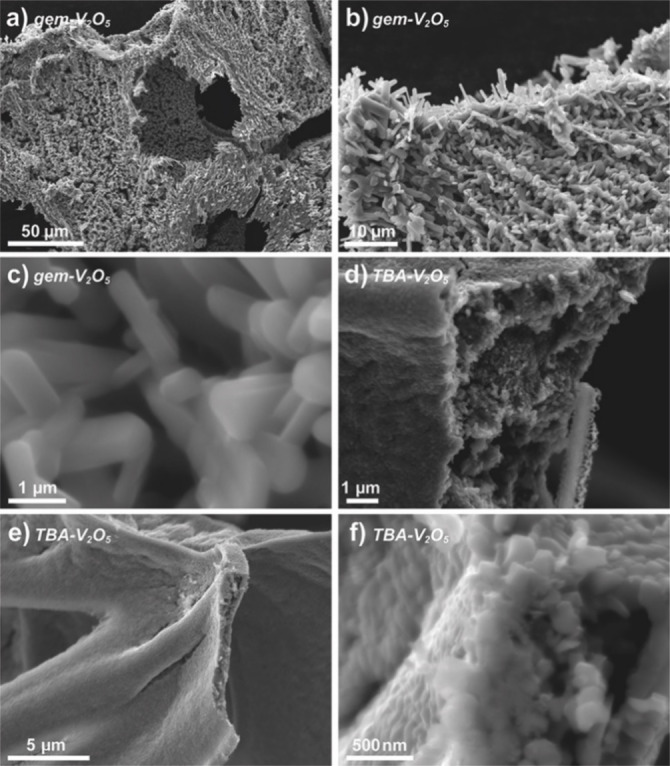
SEM images of the calcined products (550 °C, 2 h, in air): a)–c) ***gem***‐**V_2_O_5_** and, d)–f) **TBA‐V_2_O_5_**.

**TBA‐V_10_** was calcined under identical conditions to the nanostructured hybrid precursor ***gem*** **‐V_10_** (550 °C for 2 hours) to yield an apparently similar orange‐brown powder as the product (**TBA‐V_2_O_5_**). PXRD analysis finds that the product is, as in the case of ***gem*** **‐V_2_O_5_**, phase‐pure orthorhombic V_2_O_5_. Most importantly however, SEM analysis of **TBA‐V_2_O_5_** showed that the calcined product shows significantly different morphological features to those of ***gem*** **‐V_2_O_5_** (Figure 1d–f). Here, rather than the open, sponge‐like microcrystalline structure observed in ***gem*** **‐V_2_O_5_**_,_ **TBA‐V_2_O_5_** possesses a much more tightly agglomerated structure, composed of smaller and more rounded crystallites, roughly 0.5–1 μm in diameter (Figure 1f). This clear difference in morphology, despite both samples having the same crystallographic identities, is remarkable in that it clearly demonstrates a structure‐directing effect which is translated from the self‐assembled soft‐material, through to the calcination and crystal‐growth process. The ***gem*** **‐V_2_O_5_** and **TBA‐V_2_O_5_** calcination products displayed interlayer spacing of 0.53±0.11 nm (Figures S5&S6) which is in the typical range reported for V_2_O_5_.[^40^, ^41^] The nanostructure and morphology of V_2_O_5_ materials has been reported to influence electrochemical characteristics.[^42^, ^43^] Thus, we explored the impact of material morphology on the performance of the ***gem‐V***_***2***_ ***O***_***5***_ and **TBA‐V_2_O_5_** as intercalation materials in lithium‐ion batteries.

Electrodes were fabricated on aluminium foil through hand mixing of active material, conductive carbon, and binder (further details of electrode preparation can be found in the Supporting Information). Cyclic voltammetry (CV) was used to investigate the single lithium insertion/ de‐insertion behaviour within a potential window of 2.5–4.0 V vs. Li^+^/Li (Figure 2a). This reversible single‐ion intercalation has a theoretical capacity of 147 mAh g^−1^ and proceeds via a two‐step mechanism described in Equations 1 and 2.
(1)

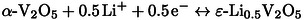



(2)

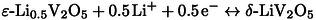




**Figure 2:**
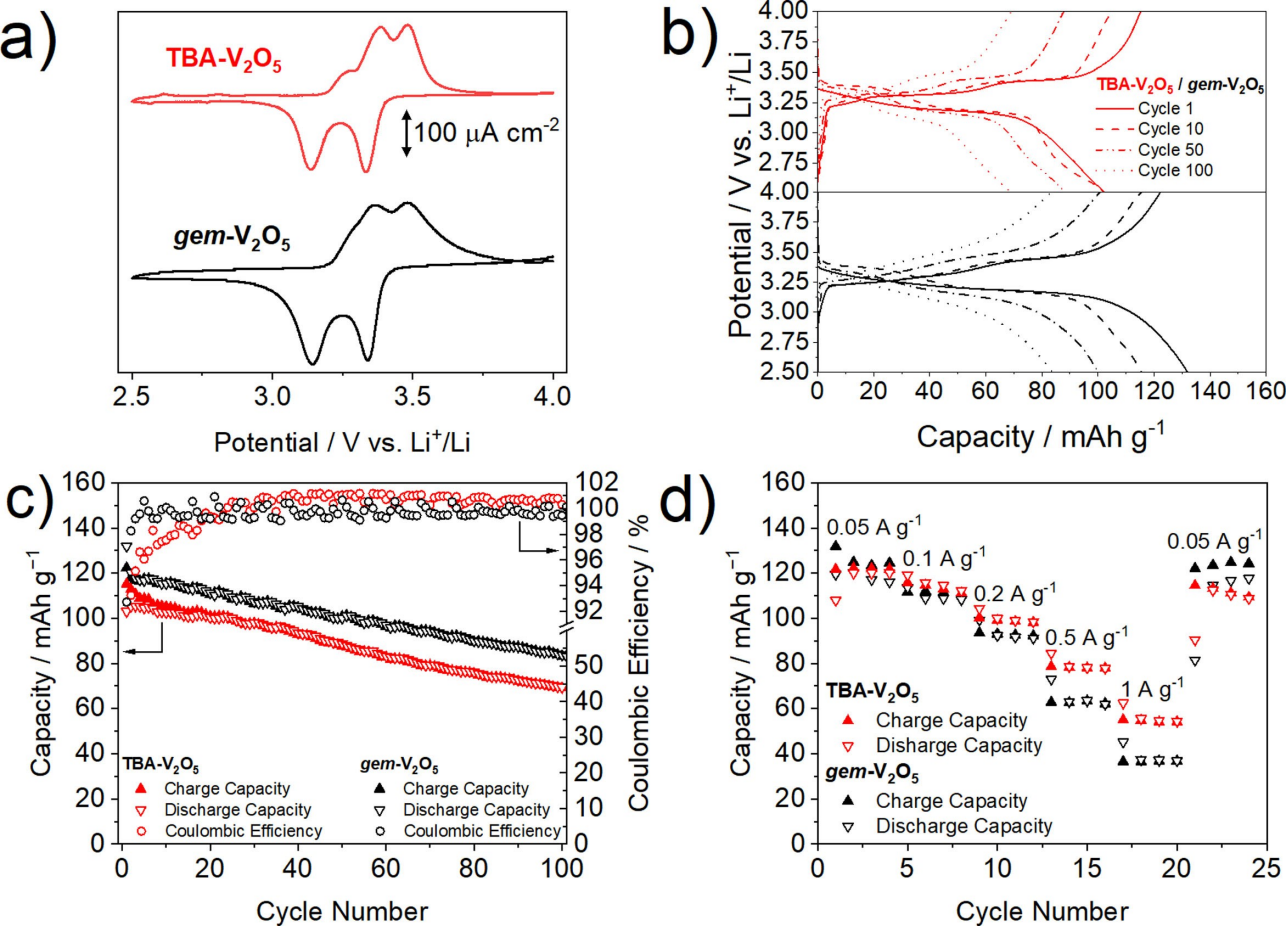
a) Cyclic voltammetry, b) voltage profiles, c) capacity performance and coulombic efficiency, and d) rate performance of electrodes cast with **TBA‐V_2_O_5_** (a), (b) red lines and (c), (d) red triangles and circles, and ***gem*** **‐V_2_O_5_** (a), (b) black lines, (c), (d) black triangles and circles.

Both ***gem*** **‐V_2_O_5_** and **TBA‐V_2_O_5_** show two pairs of well‐defined peaks that correspond to the first [Eq. (1)] and second [Eq. (2)] stages of single lithium insertion.[^16^, ^44^] This is further evidenced by two distinct plateau regions in the first cycle voltage profiles of both materials (Figure 2b).

Figure 2c shows the cycling performance and coulombic efficiency of the ***gem*** **‐V_2_O_5_** and **TBA‐V_2_O_5_** electrodes at a current density of 50 mA g^−1^, demonstrating initial lithium insertion capacities of 131.9 and 115.2 mAh g^−1^, respectively. After 100 cycles, the capacity of the materials had decreased to 83.8 and 69.2 mAh g^−1^, corresponding to a capacity retention of 63.5 and 60.0 %, respectively (Figure 2b). After the 24^th^ cycle, the **TBA‐V_2_O_5_** maintains a coulombic efficiency >100 %, with an average coulombic efficiency of 100.7 % between cycle 25–100, indicating that there is a capacity contribution from degradation reactions outside of the normal lithium insertion/de‐insertion processes.^[45]^

The ***gem*** **‐V_2_O_5_** material maintained a high coulombic efficiency of 99.63 % across the first 100 cycles. The single lithium insertion rate performance of these materials was studied using current densities between 0.05–1.00 A g^−1^ (Figure 2d). Both materials show similar rate performance up to 0.1 A g^−1^, after which the performance of ***gem*** **‐V_2_O_5_** rapidly fades compared to the **TBA‐V_2_O_5_** material. Stable capacities of ca. 36.7 and 54.2 mAh g^−1^ were recorded at 1 A g^−1^ for these materials, respectively. The capacity retention at 1 Ah g^−1^ was 28.2 and 44.5 %, compared to the initial capacity obtained at 0.05 A g^−1^, for the ***gem*** **‐V_2_O_5_** and **TBA‐V_2_O_5_** materials, respectively (Figure 2d). The difference in performance of the two materials can be explained through examining their microstructures. The ***gem*** **‐V_2_O_5_** material possesses a well‐ordered microstructure consisting of large uniform crystallites, which explains the superior capacity performance. Larger crystallites intrinsically reduce the surface‐electrolyte exposure, with a higher proportion of V_2_O_5_ being present in the protected bulk‐phase. Conversely, the smaller crystallite size, and therefore increased interfacial area, of the **TBA‐V_2_O_5_** material results in increased surface‐electrolyte exposure which enables more parasitic degradation.^[46]^ The difference in the rate performance can be similarly rationalised, with the smaller **TBA‐V_2_O_5_** crystallites facilitating faster lithium insertion kinetics as a result of higher surface area and shorter bulk migration pathways compared to the nanostructured ***gem*** **‐V_2_O_5_** material (Table S1).[^16^, ^47^] These results confirm the electrochemical structure‐performance relationship of V_2_O_5_ materials applied as lithium‐ion intercalation materials, where the capacity retention and rate performance can be rationalised as a function of crystallite size, morphology, and uniformity.

We have shown that gemini surfactant cations spontaneously self‐assemble with decavanadate clusters to yield soft nanostructured vanadium oxide (***gem*** **‐V_10_**). Furthermore, pyrolysis of these nanostructured aggregates results in the formation of highly crystalline V_2_O_5_ microstructures. Where the structure‐directing surfactant cations are replaced by alkylammonium cations (**TBA‐V_10_**), an amorphous structure is formed with no notable long‐range nanostructure which, upon pyrolysis, creates a more tightly agglomerated structure, composed of smaller, more rounded crystallites. Both pyrolysis products were confirmed to be phase‐pure orthorhombic V_2_O_5_ through PXRD analysis. The electrochemical properties of these materials were examined to determine their performance as lithium‐ion intercalation materials and distinct differences in capacity and rate performance were observed. The large crystallites and long‐range nanostructure of the ***gem*** **‐V_2_O_5_** cathode resulted in a higher initial capacity and superior capacity retention for the single lithium insertion process compared to the **TBA‐V_2_O_5_** material. The **TBA‐V_2_O_5_** material was shown to have a greater rate performance than the ***gem*** **‐V_2_O_5_** material because of the smaller crystallite size, and therefore higher surface area, enabling faster lithium insertion kinetics. The tuneable morphology, and therefore the contrasting electrochemical properties, of the V_2_O_5_ systems is proposed to arise from the structing‐directing effect of the gemini surfactant. This simple approach to the preparation of tuneable V_2_O_5_ has great potential for further development and optimisation given the vast potential array of accessible POM‐cation nanostructures.

## Conflict of interest

The authors declare no conflict of interest.

## Supporting information

As a service to our authors and readers, this journal provides supporting information supplied by the authors. Such materials are peer reviewed and may be re‐organized for online delivery, but are not copy‐edited or typeset. Technical support issues arising from supporting information (other than missing files) should be addressed to the authors.

Supporting Information

## Data Availability

The data that support the findings of this study are available in the Supporting Information of this article.
