# Supplementary material for: Self‐Assembled Surfactant‐Polyoxovanadate Soft Materials as Tuneable Vanadium Oxide Cathode Precursors for Lithium‐Ion Batteries
Source: Angew Chem Int Ed Engl. 2023 Feb 9;62(12):e202216066. doi: 10.1002/anie.202216066 (PMC10962574; doi:10.1002/anie.202216066)
Supplement: Supplementary file 1 — Supporting Information [file ANIE-62-0-s001.pdf]

Angewandte Chemie
Eine Zeitschrift der Gesellschaft Deutscher Chemiker

Supporting Information

Self-Assembled Surfactant-Polyoxovanadate Soft Materials as Tuneable Vanadium Oxide Cathode Precursors for Lithium-Ion Batteries

R. C. McNulty, K. Penston, S. S. Amin, S. Stal, J. Y. Lee, M. Samperi, L. Pérez-García, J. M. Cameron, L. R. Johnson, D. B. Amabilino*, G. N. Newton**

Experimental

1,3-Bis[(3-octacdecyl-1-imidazolio)methyl]benzene dibromide (***gem***), $K_3[H_3V_{10}O_{28}]$ (**K-V_{10}**) and $(TBA)_3[H_3V_{10}O_{28}]$ (**TBA-V_{10}**) were synthesised according to literature methods.[1,3]

Synthesis of (1,3-bis[(3-octacdecyl-1-imidazolio)methyl]benzene)$_3[V_{10}O_{28}]$ (***gem***-**V_{10}**)

$K_3[H_3V_{10}O_{28}]$ (36 mg, 33.4 µmol) was dissolved in deionised water (4.5 mL). 1,3-Bis[(3-octacdecyl-1-imidazolio)methyl]benzene dibromide ($[C_{50}H_{88}N_4Br_2]$)(45 mg, 49.7 µmol) was dissolved in EtOH (0.5 mL) and added to the previous solution slowly upon stirring for 5 minutes. The resulting suspension was then centrifuged and dried to give *gem*-V_{10} $[C_{50}H_{88}N_4]_3[V_{10}O_{28}]$ as an orange powder (82.1 mg, 25.7 µmol, 52%). ν_{max} (ATR)/cm^{-1} 3369, 3050, 2917, 2849, 1558, 1466, 946, 817, 739, 588. Elemental Analysis calc (%) for $[C_{50}H_{88}N_4]_3[V_{10}O_{28}]$: C 56.42, H 8.33, N 5.26, found: C 56.72, H, 8.81, N, 4.91.

Synthesis of ***gem***-**V_2O_5**

gem-**V_{10}** (148.8 mg, 46.6 µmol) paste was placed in an alumina boat (7.5 mL) and rested at opening of an open tube furnace (i.e. under air), set at 550 °C for two minutes. The sample was then transferred into the centre of the furnace for a further 2 hours to give the product *gem*-V_2O_5 (11.9 mg, 65.4 µmol) as an orange-brown powder. ν_{max} (ATR)/cm^{-1} 1045, 954.

Synthesis of **TBA-V_2O_5**

TBA-V_2O_5 was prepared by pyrolysis of $(TBA)_3[H_3V_{10}O_{28}]$ following the method described for ***gel***-**V_2O_5** above.

Preparation of V_2O_5 electrodes

A slurry was prepared by hand mixing V_2O_5, Super P conductive carbon, and polyvinylidene fluoride in a weight ratio of 8:1:1 with N-methyl pyrrolidone. The slurry was cast onto an aluminium sheet using an automatic doctor-blade to obtain electrode sheets. The loading amount varied from 0.8 – 1.5 mg cm^{-2}. The electrodes were dried at 120 °C under vacuum for 12 h and moved to an argon filled glovebox without atmospheric exposure.

Methods

Fourier transform infra-red (FTIR) spectroscopy. Infra-red spectra were measured using a Bruker Alpha FTIR spectrometer with a platinum ATR module.

Scanning electron microscopy (SEM). Scanning electron microscopy (SEM) was performed using a Zeiss EVO 10 MA SEM (Carl Zeiss, Germany), at an accelerating voltage of 15 kV. Energy dispersive X-ray (EDX) analysis was performed with an EDAX Elements EDX detector (AMETEK, United States of America) at an accelerating voltage of 15 kV.

Transmission Electron Microscopy (TEM). TEM imaging was performed using a JEOL 2100F FEG transmission field electron microscope (field emission gun source, information

limit 0.19 nm) operating at an accelerating voltage of 200 kV. TEM samples were prepared by drop-casting several drops of sample onto copper mesh TEM grid mounted with a lacey carbon support and graphene oxide film. Samples were dried under high vacuum unless stated otherwise

Powder X-ray Diffraction (XRD). Data was collected on a PANalytical X'Pert Pro diffractometer equipped with monochromated Cu $K\alpha_1$ radiation (λ = 1.54 Å). The tube voltage and current were 40 kV and 40 mA, respectively. Scans were performed from 5° to 70° with a step size of 0.01313 and a step time of 0.08 s/step on a zero-background silicon crystal plate.

Electrochemical Analysis. Battery cells were assembled inside an argon filled glovebox (MBraun, O_2 and H_2O ≤ 0.1 ppm). All measurements were carried out at 20 °C with an initial equilibration time of 1 hr allowed for electrode wetting and temperature uniformity. Whatman glass microfiber filter paper functioned as the separator, soaked in 100 µL of LP30 (1 M $LiPF_6$ in EC:DMC = 50:50 v/v). Li-V_2O_5 half-cells pairing 12 mm electrodes were employed for battery cycling experiments. Cyclic voltammetry was carried out in 3-electrode Swagelok-type cells consisting of a 12 mm V_2O_5 working electrode, a 12 mm lithium metal counter electrode, and a 6mm lithium metal reference electrode. All electrochemical measurements were performed on an Ivium Octostat30.

Brunauer-Emmett-Teller (BET) Surface Area Analysis. 5-point BET analysis was carried out for both the pre-calcination and calcined materials. >100 mg of sample was transferred to the measurement tube which was then degassed for 16 hours at 105 °C. The degassed sample was characterised using a Micrometrics Physisorption Analyser (3Flex) at 77 K with N_2 as the adsorptive gas.

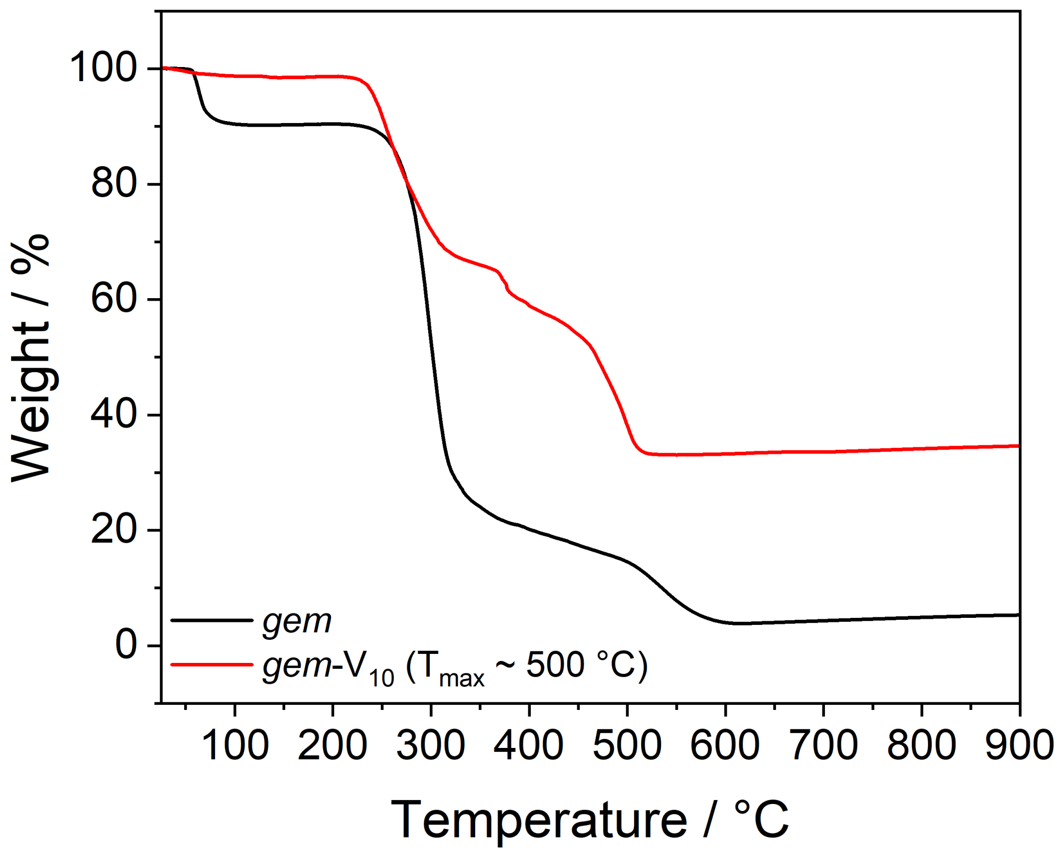

Figure S1. Thermogravimetric analysis of ***gem*-V$_{10}$**.

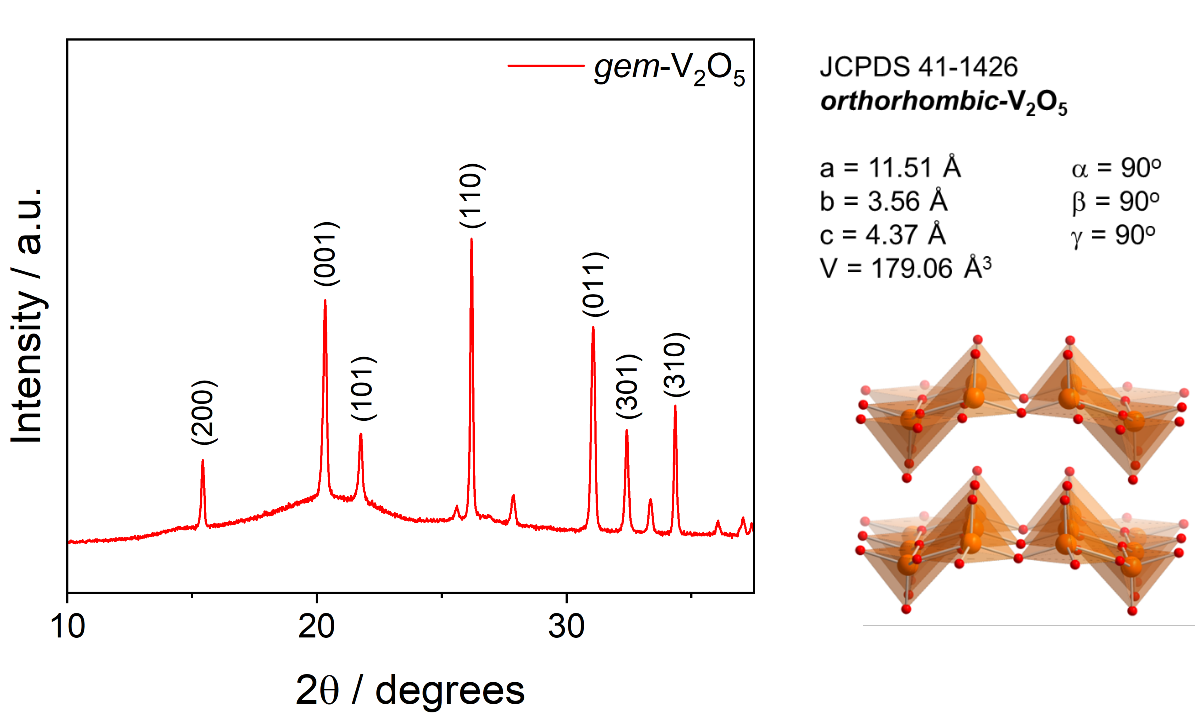

Figure S2. Powder X-ray diffraction pattern of ***gem*-V$_2$O$_5$**, indexed to that of orthorhombic V_2O_5 (structure shown on right).

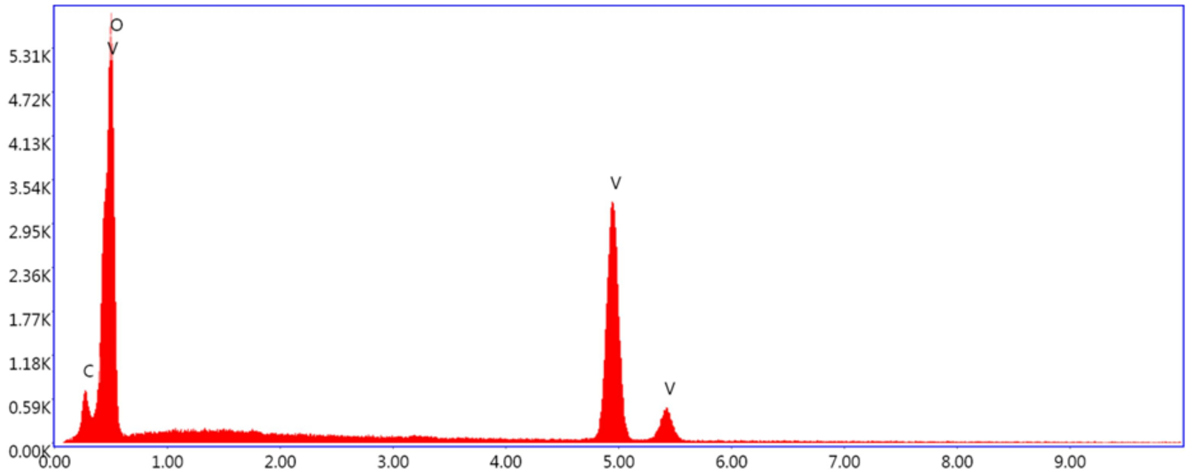

Element	Weight %	Atomic %	Net Int.	Error %	Kratio	Z	A	F
**C K**	5.81	14.70	145.98	9.52	0.0297	1.2581	0.4065	1.0000
**O K**	22.29	42.36	275.23	12.36	0.0371	1.2026	0.1384	1.0000
**V K**	71.91	42.93	1444.01	2.56	0.6562	0.9066	1.0050	1.0017

Figure S3. Energy dispersive X-ray spectrum of ***gem*-V_2O_5** (top) and quantitative EDX analysis (below). Note that the small quantity of carbon detected in the sample originates from the adhesive carbon tape used on the sample holder.

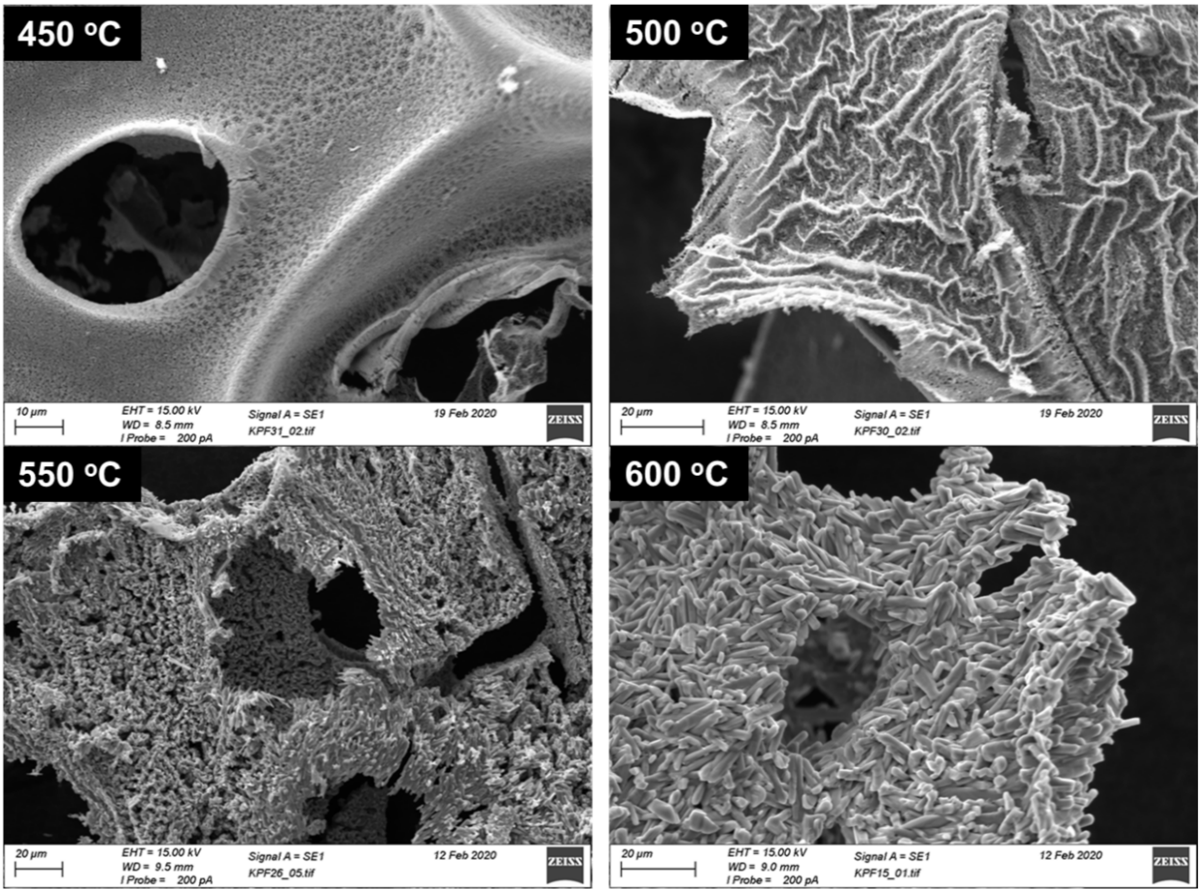

Figure S4. SEM imaging highlighting the temperature dependent crystal growth of ***gem*-V_2O_5** from 450 °C to 600 °C over 2 hours heating time.

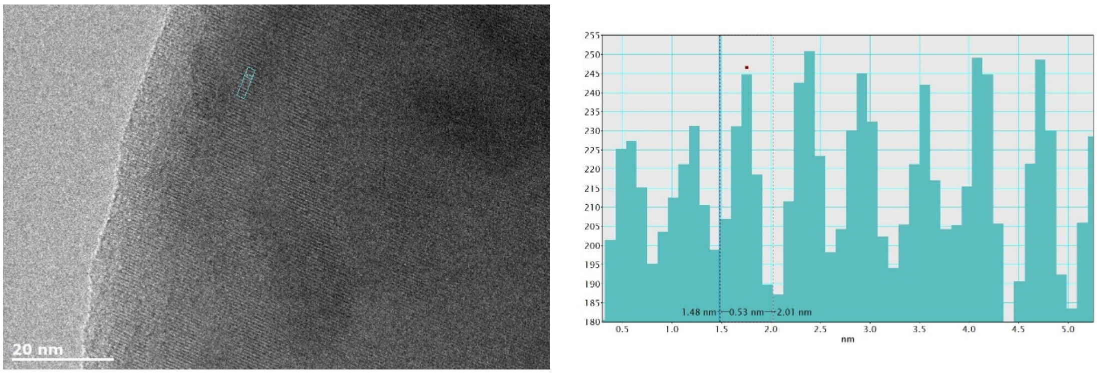

Figure S5. Transmission electron micrographs displaying the measured interlayer spacing (0.53 nm) for ***gem*-V_2O_5.**

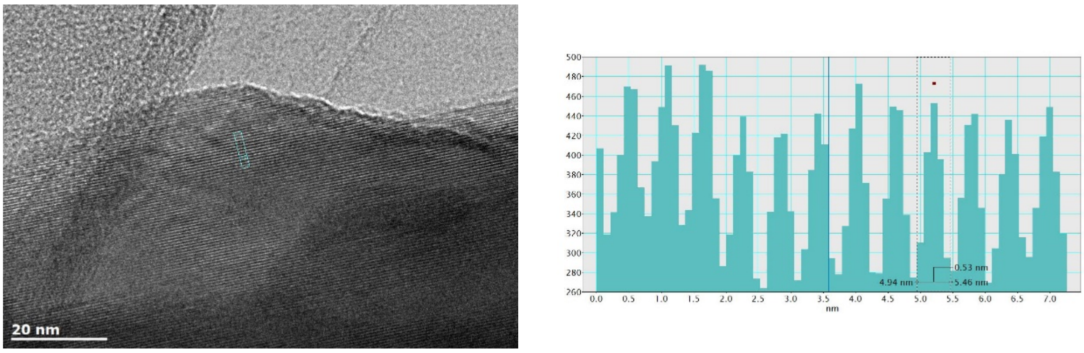

Figure S6. Transmission electron micrographs displaying the measured interlayer spacing (0.53 nm) for **TBA-V_2O_5.**

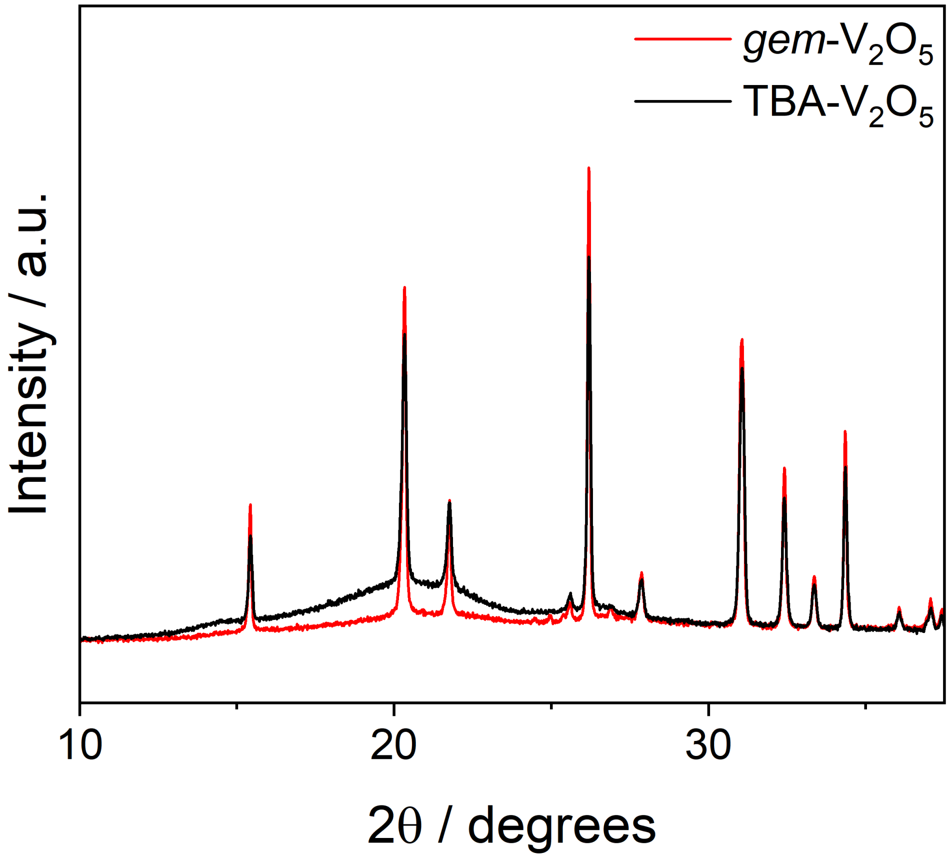

Figure S7. Powder X-ray diffraction spectra comparing the calcined products ***gem*-V_2O_5** and **TBA-V_2O_5**, both of which correspond to bulk orthorhombic V_2O_5 (as in Figure S2, above)

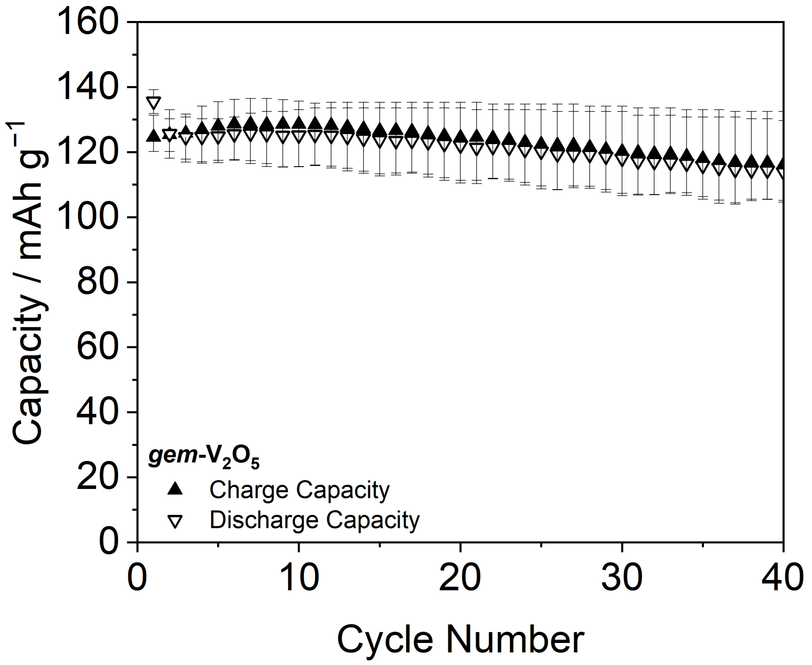

Figure S8. Average capacity performance of *gem*-V_2O_5 cells with error bars showing the absolute range of values recorded across three measurements. The central data points represent the calculated average capacities across datasets for the first 40 cycles.

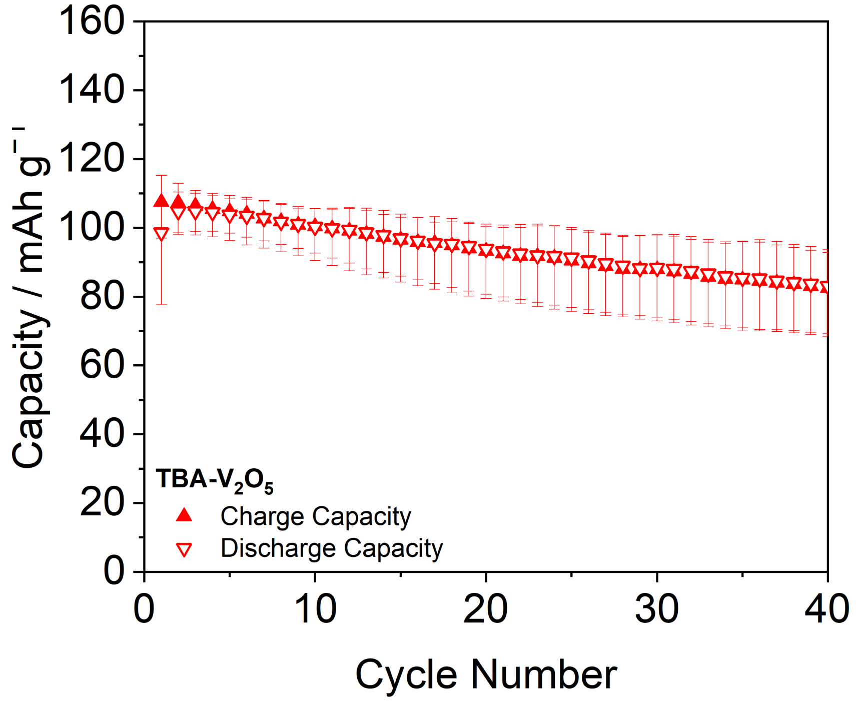

Figure S9. Average capacity performance of TBA-V_2O_5 cells with error bars showing the absolute range of values recorded across three measurements. The central data points represent the calculated average capacities across datasets for the first 40 cycles.

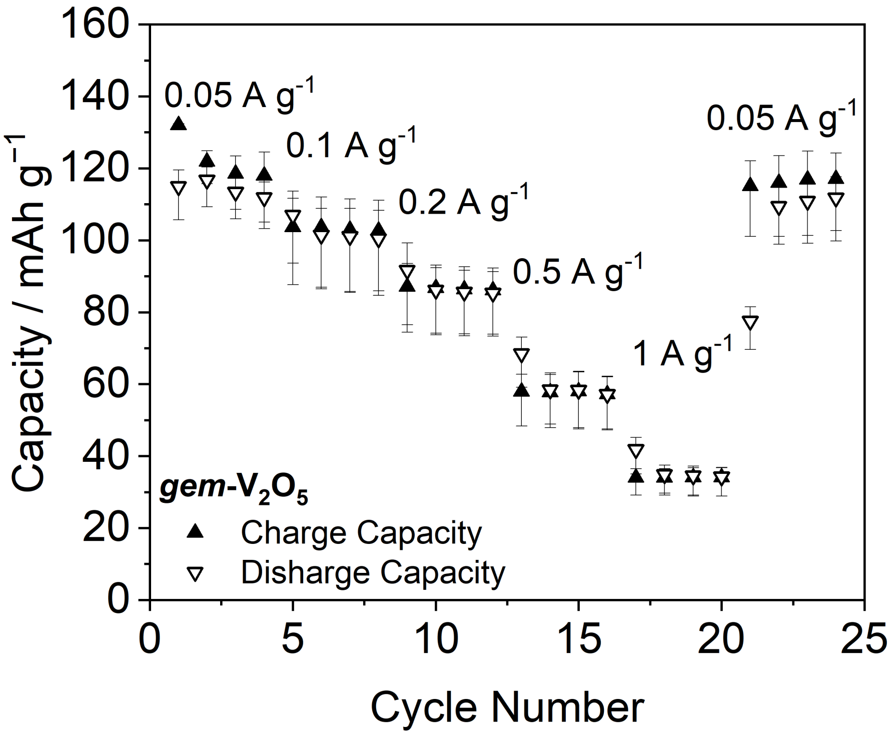

Figure S10. Rate performance of *gem*-V_2O_5 cells with error bars showing the absolute range of values recorded across three measurements. The central data points represent the calculated average capacities.

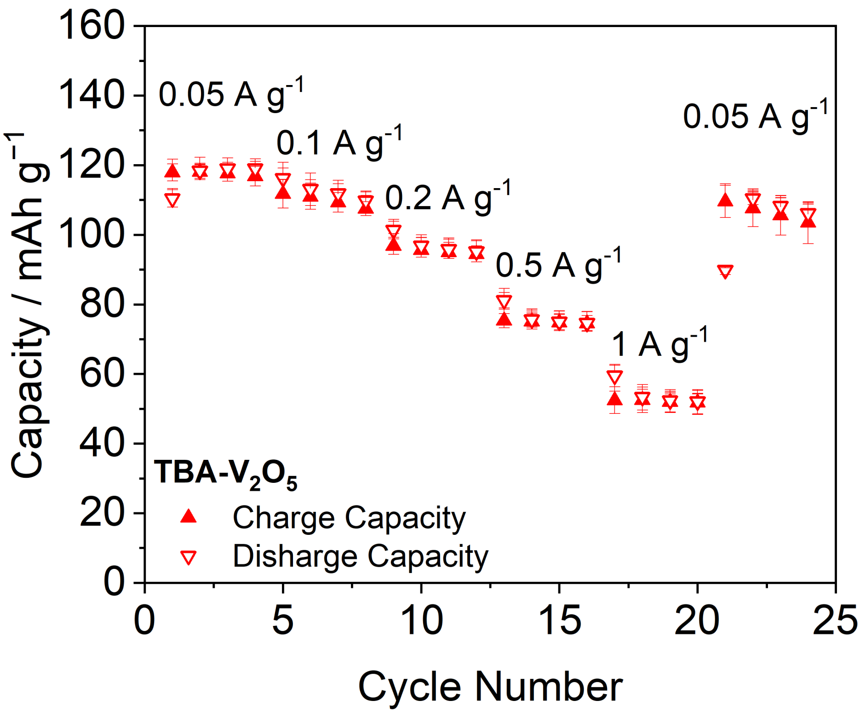

Figure S11. Rate performance of TBA-V_2O_5 cells with error bars showing the absolute range of values recorded across three measurements. The central data points represent the calculated average capacities.

Table S1. Calculated 5-point BET surface area of pre-calcined and calcined materials

Material	BET surface area ($m^2\ g^{-1}$)
TBA-V_{10}	6.177
TBA-V_2O_5	7.476
gem-V_{10}	0.648
gem-V_2O_5	2.035
